# Evidence for West Nile Virus and Usutu Virus Infections in Wild and Resident Birds in Germany, 2017 and 2018

**DOI:** 10.3390/v11070674

**Published:** 2019-07-23

**Authors:** Friederike Michel, Michael Sieg, Dominik Fischer, Markus Keller, Martin Eiden, Maximilian Reuschel, Volker Schmidt, Rebekka Schwehn, Monika Rinder, Sylvia Urbaniak, Kerstin Müller, Martina Schmoock, Renke Lühken, Patrick Wysocki, Christine Fast, Michael Lierz, Rüdiger Korbel, Thomas W. Vahlenkamp, Martin H. Groschup, Ute Ziegler

**Affiliations:** 1 Friedrich-Loeffler Insitut (FLI), Federal Research Institute for Animal Health, Institute of Novel and Emerging Infectious Diseases, Südufer 10, D-17493 Greifswald-Insel Riems, Germany; 2 German Centre for Infection Research (DZIF), Partner Site Hamburg-Luebeck-Borstel, 17493 Greifswald-Insel Riems, Germany; 3 Institute of Virology (Faculty of veterinary medicine), Leipzig University, An den Tierkliniken 29, D-04103 Leipzig, Germany; 4 Clinic for Birds, Reptiles, Amphibians and Fish, Justus Liebig University Giessen, Frankfurter Straße 91, D-35392 Giessen, Germany; 5 Clinic for Small Mammals, Reptiles and Birds, University of Veterinary Medicine Hannover, Foundation, Bünteweg 9, D-30559 Hannover, Germany; 6 Clinic for Birds and Reptiles (Faculty of veterinary medicine), Leipzig University, An den Tierkliniken 17, D-04103 Leipzig, Germany; 7 Seehundstation Nationalpark-Haus Norden-Norddeich, Dörper Weg 24, D-26506 Norden, Germany; 8 Clinic for Birds, Small Mammals, Reptiles and Ornamental Fish, Centre for Clinical Veterinary Medicine, Ludwig Maximilians University Munich, Sonnenstraße 18, D-85764 Oberschleißheim, Germany; 9 Birds of Prey Rehab Center Rhineland (Greifvogelhilfe Rheinland)/Tierarztpraxis Sudhoff, Hehnerholt 105, D-41069 Mönchengladbach, Germany; 10 Department of Veterinary Medicine, Small Animal Clinic, Freie Universität Berlin, Oertzenweg 19 b, D-14163 Berlin, Germany; 11 Wildpark Schwarze Berge GmbH & Co. KG, Am Wildpark 1, D-21224 Rosengarten, Germany; 12 Tiermedizin am Rothenbaum, Rothenbaumchaussee 195, D-20149 Hamburg, Germany; 13 Bernhard-Nocht-Institute for Tropical Medicine, WHO Collaborating Centre for Arbovirus and Hemorrhagic Fever Reference and Research, Bernhardt-Nocht Straße 74, D-20359 Hamburg, Germany; 14 Friedrich-Loeffler-Institut (FLI), Federal Research Institute for Animal Health, Institute of Epidemiology, Südufer 10, D-17493 Greifswald-Insel Riems, Germany

**Keywords:** wild bird, West Nile virus, Usutu virus, Germany, monitoring

## Abstract

Wild birds play an important role as reservoir hosts and vectors for zoonotic arboviruses and foster their spread. Usutu virus (USUV) has been circulating endemically in Germany since 2011, while West Nile virus (WNV) was first diagnosed in several bird species and horses in 2018. In 2017 and 2018, we screened 1709 live wild and zoo birds with real-time polymerase chain reaction and serological assays. Moreover, organ samples from bird carcasses submitted in 2017 were investigated. Overall, 57 blood samples of the live birds (2017 and 2018), and 100 organ samples of dead birds (2017) were positive for USUV-RNA, while no WNV-RNA-positive sample was found. Phylogenetic analysis revealed the first detection of USUV lineage Europe 2 in Germany and the spread of USUV lineages Europe 3 and Africa 3 towards Northern Germany. USUV antibody prevalence rates were high in Eastern Germany in both years. On the contrary, in Northern Germany, high seroprevalence rates were first detected in 2018, with the first emergence of USUV in this region. Interestingly, high WNV-specific neutralizing antibody titers were observed in resident and short-distance migratory birds in Eastern Germany in 2018, indicating the first signs of a local WNV circulation.

## 1. Introduction

Wild birds, especially migratory birds, play a key role in the transport of emerging pathogens and their vectors via their major flyways into Central Europe [1,2]. Monitoring wild birds and arthropod vectors (e.g., mosquitoes, ticks, etc.) is best suited to reveal the entry of new emerging (in particular zoonotic) pathogens and is a prerequisite for the rapid implementation of public and animal health measures. In Germany, a nationwide wild and zoo bird surveillance network has been in operation for several years, focusing mainly on the zoonotic flaviviruses, West Nile virus (WNV) und Usutu virus (USUV) [3,4,5,6].

WNV is a mosquito-borne arbovirus of the family *Flaviviridae* [7] and an important zoonotic pathogen worldwide. WNV is a single-stranded RNA virus [8] circulating in an enzootic cycle between ornithophilic mosquitoes as vectors and avian host species [9,10]. Infection in birds is often subclinical, although highly susceptible species, such as birds of prey, owls, or various passerine birds (crows, jays, house sparrows) may develop fatal disease [11]. A transmission of WNV via bridging vectors (mosquitoes feeding on both avian and mammalian species) to a variety of other vertebrates is possible. Although mammals such as humans and horses develop clinical signs, they are dead-end hosts, since their viremia is insufficient to infect naïve feeding mosquitoes [12]. Among infected humans, about 20% demonstrate a clinical disease, which manifests as a flu-like illness with fever, headache, and myalgia, whereas only 1% suffer from neuroinvasive disease [13]. While most horses usually seroconvert without showing any symptoms, up to 10% may develop neurological signs [14]. Clinical cases occur mainly during the peak of mosquito activity in the summer and fall [15].

Since its first emergence in Africa, WNV has spread to almost every continent (except Antarctica) and nowadays is one of the most geographically widespread mosquito-borne arboviruses [9]. In 1999, WNV was introduced into the United States of America and spread throughout the whole continent within a few years [16]. WNV has been present in Europe since the 1960s, causing sporadic outbreaks [17]. During the last two decades, there has been an increased spread of the virus within Europe. Cases among humans and horses have occurred in several European countries, for example in Hungary, Greece, Austria, Czech Republic, and Italy [9,18,19,20,21,22].

In 2018, a total of 2083 autochthonous human WNV cases were recorded in the European Union (EU) member states and EU neighboring countries. This represents a 7.2-fold increase compared to 2017 and was linked to an unusually early start of the WNV transmission season [23,24,25]. A possible explanation for the early onset and the widespread WNV epidemic in comparison to previous years are the optimal weather conditions, with an early spring and a longer period of high temperatures during the summer [26].

Taking into account that birds are the most susceptible hosts for WNV and USUV, monitoring of migratory and resident birds for the occurrence of these flaviviruses has been conducted in Germany for more than 10 years. In former monitoring studies of wild birds performed between 2007 and 2016, neutralizing antibodies against WNV were primarily detected in (long-distance) migratory birds. On the contrary, WNV RNA was found neither in wild birds nor in mosquitoes, although WNV vectors such as *Culex * spp. are common in Germany and their susceptibility to infection with WNV has been demonstrated [27,28,29,30].

In 2018 the first WNV autochthonous cases in Germany were detected in resident wild and aviary birds (12 cases), including Eurasian Blackbirds (*Turdus merula*), Northern Goshawks (*Accipiter gentilis*) and Great Grey Owls (*Strix nebulosa*), as well as in two horses in Eastern and Southeastern Germany. Chrono-phylogenetic analyses indicated a single introduction of WNV lineage 2 into Germany as early as 2016 [31].

Usutu virus (USUV) is a mosquito-borne flavivirus, closely related to WNV. It was introduced into Europe from Africa, and at first caused initially unrecognized deaths in birds in 1996 in Italy [32,33]. In 2001, the first large European outbreak occurred in Austria, causing a massive die-off among Eurasian Blackbirds and other bird species [34,35,36]. In Germany, USUV emerged in 2011 in the region of the Upper Rhine valley, where it had previously been detected in a mosquito one year before. This led to a massive reduction of the local blackbird population [37,38]. During the following years, USUV outbreaks remained geographically restricted to the Upper Rhine Valley in Southwest Germany, apart from a few sporadic cases in Berlin and Bonn [6,39,40]. In 2016, however, case numbers increased dramatically not only in Southwestern Germany, but also in the federal states of North Rhine-Westphalia, Saxony, and Saxony-Anhalt (region of Leipzig and Halle) [41,42].

USUV isolates can be phylogenetically differentiated into eight different lineages: Africa 1–3 and Europe 1–5 [32]. Four of them (Europe 3, Europe 5, Africa 2, and Africa 3) are circulating in Germany [41,42]. USUV is considered to have a very low zoonotic potential, and infections in humans are usually asymptomatic. Nevertheless, in recent studies, a neuroinvasive disease was occasionally detected in humans, however, mainly in immunocompromised patients [43,44]. USUV was also detected among healthy blood donors and therefore may poses an underestimated threat for blood recipients [44,45,46].

The massive spread of USUV and the first WNV detection in Germany in 2018 emphasize the importance of country-wide monitoring studies. Our serological and molecular results in migratory and resident birds in Germany sampled in 2017 and 2018, highlight the contribution of laboratory monitoring in aiding risk assessment for zoonotic flaviviruses.

## 2. Materials and Methods 

### 2.1. Sample Collection

**Figure 1:**
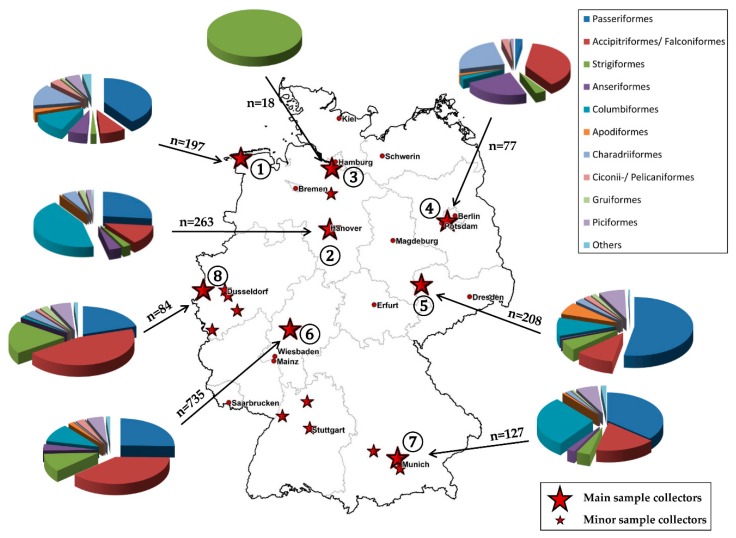
Total number of samples (first sample panel) collected in 2017 and 2018 per sampling site divided into the different zoological bird orders (big red stars = main sample collectors, small red stars = minor sample collectors). The encircled numbers next to the sample collectors allow a geographical allocation to the respective sample collector: ① “Seehundstation Nationalpark-Haus Norden-Norddeich”; ②“Clinic for Small Mammals, Reptiles, and Birds of the University of Veterinary Medicine Hannover”; ③ “Wildpark Schwarze Berge, Rosengarten”; ④ “Department of Veterinary Medicine, Small Animal Clinic of the Freie Universität Berlin; ⑤ “Clinic for Birds and Reptiles (Faculty of Veterinary Medicine) of the Leipzig University”; ⑥ “Clinic for Birds, Reptiles, Amphibians and Fish of the Justus Liebig University Giessen”; ⑦ “Clinic for Birds, Small Mammals, Reptiles and Ornamental Fish, Centre for Clinical Veterinary Medicine of the Ludwig Maximilians University Munich”; ⑧ “Birds of Prey Rehab Center Rhineland”.

Wild bird blood samples were collected through the nationwide wild bird surveillance network for zoonotic arthropod-borne viruses, including clinics or institutes of the veterinary universities, bird clinics, wild bird rescue stations, zoological gardens or wildlife parks, and falconries distributed all over Germany. We were able to expand our monitoring program with new sample collectors, especially in (new) USUV hotspot regions and by including contributors in the Eastern part of Germany. The geographical location of the different sampling sites and the investigated wild birds grouped into bird orders are shown in Figure 1. Major contributors are marked with big red stars. We also received wild bird blood samples from a number of smaller bird clinics and falconries via the University of Giessen, which are depicted as small red stars. The encircled numbers next to the sample collectors allow a geographical allocation of the samples to the respective sample collector. For a better interpretation of the data, we divided the sample collection sites into three geographic regions. Region A (Northern Germany) includes the sample collectors’ ① to ③. From the “Wildpark Schwarze Berge” (③) we received samples exclusively in 2018, due to a massive USUV outbreak in this region in 2018. Region B (Eastern Germany) is represented by the numbers ④ and ⑤. Region C (Central and Southern Germany) comprises the sample collectors’ ⑥ to ⑧. Via the University of Leipzig, we predominantly received samples from Saxony, but also sporadically from Baden-Wurttemberg, North Rhine-Westphalia, Lower Saxony, Mecklenburg-Western Pomerania, and Saxony-Anhalt. Therefore, these samples were assigned to the respective geographic region. Our monitoring, based on the German nationwide wild- and zoo-bird surveillance network, was mainly restricted to live birds. Birds were categorized as resident birds (remain all year in their German habitat), partial migratory birds (parts of the population stay in the German habitat and parts migrate), short-distance migratory birds (usually migrating 1000–2000 km and not passing through the Sahara desert), and long-distance migratory birds (usually migrating 3000–4000 km and/or passing the Sahara desert). In our first sample panel we also included a small number of liver samples from birds that either died or were euthanized at the Veterinary Clinic at the University of Leipzig.

The blood samples were obtained during the routine investigation of sick and injured birds, when blood was taken for hematological or blood chemical examination. Blood collection was facilitated by puncturing the wing, metatarsal, or jugular veins. After blood separation, the cruor was stored at −70 °C and the serum at −20 °C until sample processing. We recorded the weight and took radiographs of the birds to avoid re-sampling. A double sampling of the same bird is unlikely but cannot be excluded completely.

The second sample panel consisted exclusively of organ samples (brain, liver, spleen, or heart) which were submitted to the Friedrich-Loeffler-Institute (FLI) national reference laboratory for WNV by the regional veterinary laboratories of the federal states of Germany, by the German Mosquito Control Association (KABS), the Nature and Biodiversity Conservation Union (NABU), citizens and independent bird clinics, and zoological gardens. These samples were analyzed in close cooperation with the Bernhard-Nocht-Institute for Tropical Medicine in Hamburg and the Institute of Virology at the Faculty of Veterinary Medicine in Leipzig. Not all organs could be collected from every bird, so we focused on examining the brain, liver, and spleen.

### 2.2. Ethical Statement

We used blood samples from birds already at veterinary clinics or wild bird rescue centers. These birds were previously treated and under the care of veterinarians and veterinary technicians. All blood samples were surplus from those taken initially for diagnostics, complete blood counts, and chemistry panels. We collected tissues only from deceased birds.

### 2.3. Quantitative Real-Time Polymerase Chain Reaction (qRT-PCR) and Phylogenetic Analysis

Viral RNA of the avian blood and organ samples was extracted using the RNeasy Mini Kit (Qiagen, Hilden, Germany), according to the manufacturer′s instructions. The extracted RNA was amplified using a WNV-specific real-time polymerase chain reaction (qRT-PCR), which ensures the detection of lineage 1 and 2 [47]. Furthermore, the samples were tested using the USUV-specific qRT-PCR described by Jöst et al. [37]. Cycle threshold (Ct) values below 37 were regarded as positive, from 37 to 40 as suspicious, and above 40 as negative. Samples with Ct values above 37 in the USUV qRT-PCR were additionally analyzed using the primers and probes described by Cavrini et al. [48].

Sequencing was performed on a partial segment of the envelope USUV gene by the University of Leipzig and the Friedrich-Loeffler-Institute (FLI), however, with a small overlapping region of only 474 nucleotides. Therefore, two phylogenetic trees were built based on alignments of 1066 nt, and 726 nt of the envelope-coding gene regions in MrBayes 3.2.6. A Bayesian Monte Carlo Markov Chain (MCMC) method and in parallel a maximum likelihood (ML) tree with 1000 bootstrap replicates was used to build a consensus tree [39,49]. The detected USUV sequences were aligned to already published sequences using the MAFFT alignment. The sequence of lineage Africa 1 was used as an outgroup to root the trees. Only bootstrap values above 80 are shown. Sequencing was not always possible in samples with poor quality, this was mostly associated with higher Ct values above 36 in organs.

### 2.4. Serological Investigations 

From the 1709 wild bird blood samples we obtained 1533 sera. In the absence of a suitable ELISA for detection of WNV and USUV antibodies in birds requiring only small sample volumes (<50 µl), and an ELISA with a high sensitivity and specificity against the serogroup cross-reactivity with other flaviviruses, we decided to investigate all serum samples by virus neutralization tests (VNT). The serum samples were investigated against the WNV strain Austria (acc. no. HM015884, kindly provided by S. Revilla-Fernandez, AGES Mödlingen, Austria) and the USUV strain Germany (acc. no. HE599647) in VNTs to identify the cross-reacting antibody titers among the Japanese encephalitis serogroup. The virus neutralization tests were conducted as described by Seidowski et al. [3]. The neutralizing antibody titer (neutralization dose 50% (ND50)), of a serum sample was defined as the maximum dilution which inhibited cytopathic effects in fifty percent of the wells and was calculated according to the Behrens–Kaerber method. Serum samples with ND_50_ values above 10 were evaluated as positive, and samples with ND_50_ values lower than 10 as negative. Wild birds were only regarded positive for WNV if they had a negative (ND_50_ < 10) or significantly lower (two-fold lower) USUV titer. The same criteria for interpretation of VNT results was applied for USUV serology. Not all samples could be analyzed or evaluated, due to small sample volumes, cell toxicity, hemolysis, or due to the inability to differentiate between the specific antibody titers.

### 2.5. Maps

GIS (geographic information system) analysis of the different sampling sites (Figure 1) and the location of the USUV-positive birds used for sequencing (Figure 3) was performed by using the ArcGIS ArcMap 10.5 software (ESRI, Redlands, CA, USA) and © GeoBasis-DE /BKG 2017. Figure 4, depicting the total number of birds perished from USUV infection in 2017, was produced using the R software using the packages raster, maptools, and magrittr [50,51,52,53].

## 3. Results

Between 2017 and 2018, 1709 blood samples of wild birds, belonging to 19 different bird orders and 143 wild bird species (Table 1), were collected within the German nationwide wild bird surveillance network (first sample panel). Of these samples, 1607 could be analyzed in qRT-PCR and 1533 serum samples were investigated serologically.

### 3.1. Quantitative Real-Time Polymerase Chain Reaction (qRT-PCR) Results

Of 1607 wild bird samples from the first sample panel tested for WNV and USUV all were negative for WNV specific RNA, but 57 were USUV positive:

In 2017, 32 of 826 wild birds were USUV-RNA-positive (Table 2a). Most positive birds were Eurasian Blackbirds from the federal states of Saxony (*n* = 25) and several other bird species of the zoological order Passeriformes. One Great Spotted Woodpecker (*Dendrocopos major*) from Osteel (Lower Saxony) was tested USUV-positive, representing the northernmost USUV case in our live bird sample panel in 2017.

In 2018, USUV-specific RNA was found in 25 of 781 birds (Table 2b), mainly in Eurasian Blackbirds from the federal States Saxony (*n* = 6), Lower Saxony (*n* = 4), and Hesse (*n* = 3).

Furthermore, a broad spectrum of other wild and captive passerines and birds of the orders Strigiformes and Falconiformes from all over Germany were positive (Table 2).

The second set of samples consisted of organ samples from found dead or perished wild- and zoo-birds submitted to the national WNV-reference laboratory at the FLI from different institutions (for more details see Section 2.1. *Sample collection*). Among these investigated organ samples, altogether, 100 dead birds were tested USUV-positive in 2017, belonging to the bird orders Passeriformes and Strigiformes (Table 3).

### 3.2. Phylogenetic Analysis of Usutu virus (USUV)-Positive Samples

**Figure 2:**
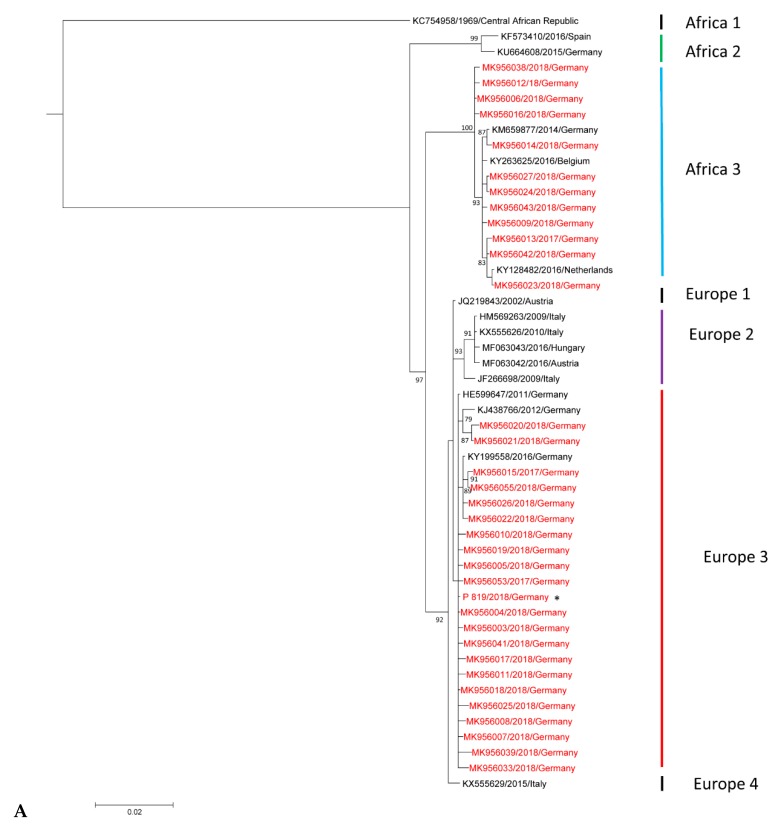

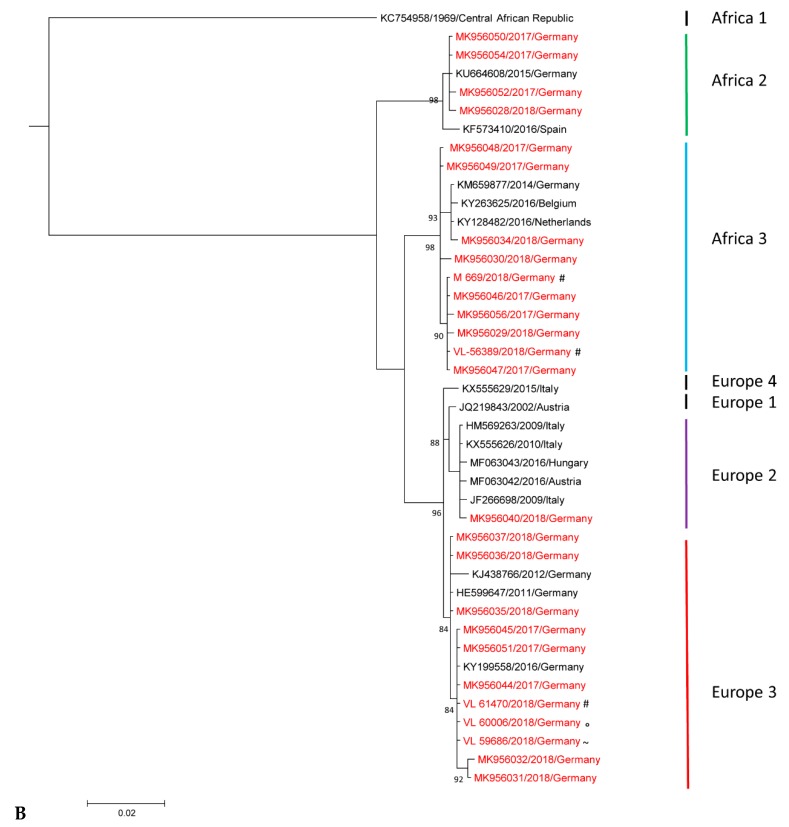
Phylogeny of the Usutu virus (USUV) isolates in 2017 and 2018. Sequences detected in 2017 and 2018 are highlighted in red. Taxon information includes the GenBank accession numbers, detection years, and countries of origin of the viruses. Scale bars indicate the mean number of nucleotide substitutions per site. Some samples had identical partial sequences, but are also depicted as they are from a different geographic region or year, respectively: (* = sequence identical to MK956004; # = sequence identical to MK956046; ° = sequence identical to MK956045; ~ = sequence identical to MK956044) (**A**) Phylogenetic tree of the detected USUV strains from Germany and Europe constructed from partial envelope genome nucleotide sequences (1066 nucleotides) sequenced by the FLI (Friedrich-Loeffler-Institute). (**B**) Phylogenetic tree of the detected USUV strains from Germany and Europe constructed from partial nucleotide sequences for the envelope protein (726 nucleotides) sequenced by the University of Leipzig.

**Figure 3:**
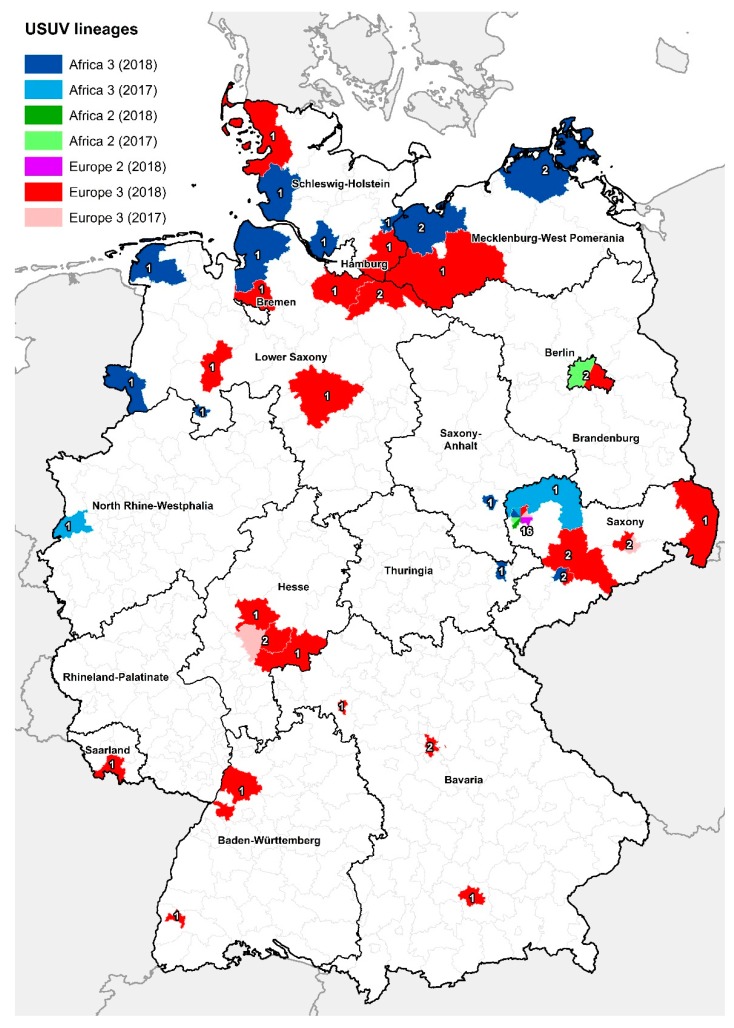
Distribution of the different USUV lineages in 2017 and 2018 (depicted on district level). For sequencing, both the birds of our live bird sample panel and the dead birds submitted to the National reference laboratory were used. The total number of birds sequenced per district is also embedded in this map.

To determine the geographic distribution of the different USUV lineages in 2017 and 2018, RNA-positive samples of the live bird samples panel (first sample panel) and of the dead birds (second sample panel) which were submitted to the national WNV-reference laboratory at the FLI from nearly all federal states of Germany were sequenced (Figure 2A,B). Whereas in Southern Germany only the lineage Europe 3 was detected, in Central and Northern Germany, additionally, the lineages Africa 2 and Africa 3 were found. In general, a massive spread of Europe 3 and Africa 3 towards the North of Germany was seen in 2018, while lineage Africa 2 was more restricted to Eastern Germany (Saxony, Saxony-Anhalt, Berlin). Furthermore, in 2018, the USUV lineage Europe 2 (included in Figure 2A) was detected for the first time in Germany, in the federal state of Saxony. Detailed information about the distribution of the different USUV lineages at district level is depicted in Figure 3. Additional information regarding the bird species, location, and USUV lineage of the respective GenBank accession numbers are portrayed in the Supplemental Table S2.

### 3.3. Serological Results

For the evaluation of the VNT results, the different samples were grouped according to their geographical origin. Overall, 1533 serum samples were assayed for WNV- and USUV-neutralizing antibodies (first sample panel). 

In 2017, 22 out of 739, and in 2018, 26 out of 794 investigated wild bird sera contained neutralizing antibodies against WNV (Table 4, Table 5 and Table 6). WNV seropositive birds were found occasionally in all geographic regions (Region A–Region C), displaying quite low neutralizing antibody titers generally ranging between ND_50_ 1:10–1:20 and sporadically up to ND_50_ 1:80. These WNV-positive birds were mainly long- and short-distance migratory birds. However, also in a few partial migrants, and only sporadically even in resident bird species, WNV neutralizing antibodies were found. Noteworthy is the detection of high antibody titers against WNV in Region B (Eastern Germany) in 2018, where also indigenous WNV cases were found for the first time in owls, birds of prey, and horses. In this region, in 2018, three birds of prey and one Carrion Crow (*Corvus corone*), showed higher neutralizing antibody titers against WNV, in contrast to the previous year, with ND_50_ values between 1:160 and 1:320 (Table 5).

USUV neutralizing antibodies were found in 38 wild birds examined in 2017 and in 67 wild bird samples in 2018. In Region A (Northern Germany) in 2017, only three wild birds showed neutralizing antibodies against USUV with relatively low titers (ND_50_ values 1:10, 1:20), whereas in 2018, 29 wild birds were seropositive with high antibody levels in this region (antibody titers ranged up to 1:2560 ND_50_ (Table 4). Neutralizing antibodies were found predominantly in Passeriformes, which are mainly resident birds or partial migratory birds, and in Strigiformes which were kept in zoological gardens. In Region B (Eastern Germany) in 2017 16, and in 2018 12, wild birds were USUV seropositive. Antibody titers ranged from 1:10 to 1:480 ND_50_ (Table 5). A broad spectrum of different bird species showed neutralizing antibodies in this region (predominantly Eurasian Blackbirds (*Turdus merula*)), but also Common Wood Pigeons (*Columba palumbus*) and Eurasian Woodcocks (*Scolopax rusticola*). These birds are primarily resident, partial or short distance migratory species. In Region C (Central and Southern Germany) 19 wild birds had neutralizing antibodies against USUV in 2017 and 26 in 2018. Samples were predominantly from Passeriformes and Columbiformes, but additionally a large number of zoo birds of the orders Accipitriformes/Falconiformes, showed neutralizing antibodies against USUV (Table 6).

In summary, predominantly resident and partial migratory birds, but also short-distance migratory birds and numerous zoo birds showed neutralizing antibodies against USUV. In Northern Germany in 2018, the number of seropositive birds and the ND_50_ values increased in comparison to the previous year, especially in areas where USUV was not detected before.

In 14 of the 1533 serum samples, a discrimination between USUV and WNV antibodies was not possible by VNT, as ND50 values were comparable for both viruses (maximum 1.5-fold differences). For detail, see Table 7. The VNT results of all wild bird species between 2017 and 2018 are presented in the Supplemental Table S1.

Furthermore, due to small sample volumes, the collection and investigation of both cruor and serum was not possible for some samples.

## 4. Discussion

Wild birds are vectors and reservoir hosts of endemic or re-emerging zoonotic pathogens and play a substantial role in their local dispersal and maintenance in Central Europe. The first detection of the WNV genome in Europe was in France in the 1960s, causing outbreaks among humans and horses [9]. After a longer period of absence, the virus reemerged with outbreaks in Ukraine (1985), Romania (1996), and Russia (1999) [12]. In the last two decades, several European countries reported a series of outbreaks of WNV neuroinvasive disease in humans and horses. The virus hit Mediterranean countries first, with a steady northward expansion. Eventually, WNV was detected in neighboring countries to Germany like France, Austria, and in the Czech Republic [54,55,56,57,58]. In Poland, high WNV seroprevalences were detected among birds and horses, also indicating WNV activity in Poland [59]. Therefore, an introduction into Germany could be expected, as WNV was already “ante portas”. [12].

WNV-specific RNA was not detected in our first sample panel of live wild- and zoo-birds collected in 2017 and 2018, albeit the first clinical cases were detected for the first time in August–October 2018, involving various bird species and horses, in the eastern and southeastern parts of Germany [31]. Negative WNV qRT-PCR results were also obtained in earlier wild bird monitoring studies from samples taken in the time period 2007 to 2016, whereas neutralizing antibodies against WNV were detected regularly in migratory birds [3,4,5]. It was therefore assumed that WNV was not yet circulating in Germany until 2016. The WNV situation in Germany is still unclear given that neutralizing antibodies are regularly found in resident non-migratory birds. However, phylogenetic analysis of the first Germany WNV strain from 2018 revealed its descendants from a Czech strain with a putative introduction into Germany already in 2016 [31].

The serological investigation revealed WNV neutralizing antibodies in 48 out of 1533 investigated wild birds in 2017 and 2018, which is quite a small number. We detected antibodies in long-distance migratory birds such as a Stork (*Ciconia* sp.), Common Swift (*Apus apus*), or Common Cuckoo (*Cuculus canorus*), but also in short-distance and partial migratory birds, as described before [5]. These birds most probably survived WNV infections at their overwintering quarters or during migration. However, neutralizing antibodies were also detected in birds of prey (bird orders Accipitriformes and Falconiformes) in 2017 and 2018. Birds of prey are considered to be highly susceptible to WNV, which might be an explanation for the detection of antibodies in these birds [60,61,62]. Nevertheless, the antibody titers were generally quite low, suggesting older infections. As reported before, a considerable number of resident birds belonging to the orders Passeriformes (Eurasian Blue Tit (*Cyanistes caeruleus*), Common Magpie (*Pica pica*)) showed neutralizing antibodies against WNV, however, the reason remains unclear. Until 2018, no local WNV circulation was detected in Germany, and it was therefore assumed that the resident birds probably became infected in neighboring countries, where WNV has been present for quite some time. Besides that, even so-called resident birds sometimes can travel about 50–100 km [1]. In 2018, high neutralizing antibody titers (ND_50_ 160–320) were found in one Northern Goshawk (*Accipiter gentilis*), one European Kestrel (*Falco tinnunculus*), one White-tailed Eagle (*Haliaeetus albicilla*), and one Carrion Crow (*Corvus corone*) in the regions of Leipzig and Berlin, possibly indicating a recent WNV infection. This correlates with previous detections of WNV-RNA in birds in the same region [31]. The first indications of WNV circulation in Germany can therefore also be seen in our wild bird monitoring of live birds of 2018. 

The closely related USUV has been present in Germany since 2010/2011 and has spread all throughout Germany over the years [38,41]. As the transmission cycles of WNV and USUV are rather similar, with wild birds as reservoirs and amplifying hosts and mosquitoes of the Culex spp. as vectors, the question arises if a similar distribution scenario is possible for WNV in the future as well [63].

**Figure 4:**
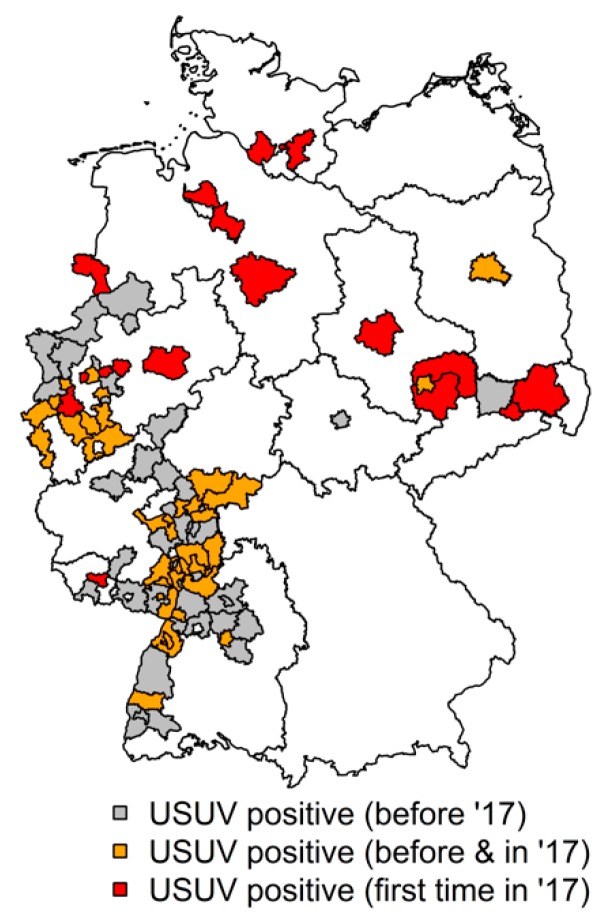
USUV detection in dead birds (second sample panel) in 2017 (red areas = USUV-positive birds detected for the first time in 2017, yellow areas = USUV-positive birds detected before and in 2017, grey areas = distribution of USUV-positive birds in the previous years, but no cases in 2017).

In 2017, USUV occurred for the first time in the regions of Hannover, Hamburg, and Bremen, indicating a further spread towards the north of Germany. Of the investigated dead birds of our second sample panel from 2017, in total, 100 birds were tested USUV-RNA-positive and negative for WNV-RNA. Detailed information on the location of the USUV-positive samples of the dead bird investigation are displayed in Figure 4. Among our first sample panel, altogether, 32 wild birds were tested USUV-RNA-positive, with one positive Great Spotted Woodpecker from Osteel (Lower Saxony), representing the northernmost USUV case in our sample panel in 2017. In both the live and the dead bird samples (first and second sample panel), a northward movement of USUV in 2017 is portrayed.

Pathogenesis studies with the closely related WNV showed that the viremia of an infected bird is only detectable for a few days, whereas in the organs, the virus persists longer, especially in birds of the order Passeriformes [64,65]. The same may apply for USUV. This might also be an explanation for the lower number of USUV-positive birds in our first sample panel, where predominantly blood samples of live birds were investigated, in comparison to the total number of USUV-positive dead birds (second sample panel), consisting exclusively of organ samples from dead-found or perished wild- and zoo-birds submitted to the national WNV reference laboratory at the FLI.

In 2018, it came to a massive USUV outbreak with thousands of dead Eurasian Blackbirds and other passerines, as well as birds of prey and various owls in aviaries. Unfortunately, as most infections occurred under natural conditions, exact numbers of infected birds are difficult to estimate. It is however already clear that in 2018, the largest USUV epizootic since 2011 was observed, with all federal states of Germany being affected [66].

The spatial expansion of USUV corresponds to high temperatures in 2018 and thereby a shorter extrinsic incubation period, resulting in a faster transmission cycle between bird host and mosquito vector and a high infection pressure, leading to a broad spectrum of infected bird species. This is reflected in the distribution of the USUV-positive birds in our live bird survey (first sample panel) of 2018, with cases located all over Germany. Apart from the most susceptible bird species like Eurasian Blackbirds and Great Grey Owls, numerous other bird species were affected, such as different bullfinch species (*Pyrrhula pyrrhula*, *Pyrrhula erythaca*), one Domestic Canary (*Serinus canaria forma domestica*), and one European Kestrel (*Falco tinnunculus*). In both the live and the dead bird monitoring studies, Eurasian Blackbirds, being the most USUV-susceptible bird species, were most often infected with USUV. This led to a massive die-off and consequently a drastic reduction of the Blackbird population. In 2011, with the first USUV infections in birds in Germany, Eurasian Blackbirds disappeared completely in certain regions. A negative impact on the Blackbird population was demonstrated in USUV outbreak areas in comparison to unsuitable USUV areas, while no such correlation was found for other bird species [67,68].

Furthermore, we sequenced USUV-positive samples from our live and dead bird survey to determine the geographic distribution of the different USUV lineages. In Germany in 2018, the USUV lineages Africa 3 and Europe 3 spread massively and were detected in numerous federal states, especially in the northern parts of Germany. USUV lineage Europe 3 was, until then, restricted to the South of Germany, but has spread all over Germany since its first discovery in the Leipzig area in 2016 [42]. The same applies for lineage Africa 3, which has most likely been introduced into Germany just recently and was first detected in Bonn in 2014 [40]. Since 2016, USUV lineage Africa 3 was also found in the federal state of Saxony. The lineage Africa 2, however, seems to be more restricted to Eastern Germany [39,42]. The co-circulation of different USUV lineages within one country was also seen in other European countries like Italy, Austria, and France [69,70,71].

Four different USUV lineages have been found in the federal state of Saxony to date, with the first occurrence of Europe 2 in 2018. Typically, Europe 2 was circulating in Austria, Hungary, and Italy for years, and its introduction via infected birds into Germany is a possible scenario [46,72]. Apart from the introduction via viremic birds, the transport of infected mosquitoes by car, ship, or plane also seems a possible option, as the first appearance of USUV in Austria was near the largest airport nationwide [68]. As mentioned above, similar single introduction events also happened in Bonn and Berlin with the entry of Africa 3 and Africa 2, respectively [38,39]. Finally, the reasons for the occurrence of multiple USUV lineages around Leipzig remains unclear.

Neutralizing antibodies against USUV were detected in 105 of 1533 investigated wild bird blood samples. Mainly affected birds were resident birds, but also some partial, and short-distance migratory birds, which most probably became infected in Germany or close neighboring countries. Neutralizing antibodies against USUV were detected in a broad spectrum of wild and zoo birds belonging to several different bird orders. However, this was also seen in previous monitoring studies, suggesting a widespread activity of USUV within Germany, affecting several bird species [5].

By comparing Northern, Eastern, Central, and Southern Germany during 2017 and 2018, differences regarding the number of USUV antibody positive birds can be noticed. In Northern Germany (Region A), in 2017, USUV antibodies were detected only sporadically in three birds (seroprevalence 1.75%) with quite low antibody titers, whereas in 2018 the number of seropositive birds increased immensely (seroprevalence 9.54%). Around Norddeich (sample collector ①) and the “Wildpark Schwarze Berge” (sample collector ③) near Hamburg, there was a massive USUV outbreak in 2018, resulting in numerous perished birds [66]. This outbreak can also be demonstrated in our live bird monitoring (first sample panel) with seropositive birds from a broad spectrum of different bird species. Furthermore, a large number of birds showed high antibody titers up to 1:2560 ND_50_, indicating a recent infection with USUV. Especially in the “Wildpark Schwarze Berge” (sample collector ③), eight out of eighteen investigated captive owls had high USUV antibody titers.

In Eastern Germany (Region B), represented by the clinics of the veterinary universities of Berlin (sample collector ④) and Leipzig (sample collector ⑤), in both years, USUV neutralizing antibodies were found in the sampled birds with titers ranging from 10 to 480 ND_50_. The seroprevalence was very high in 2017 (21.05%) and 2018 (13.04%). In the sampled regions, USUV has already been present for several years causing massive outbreaks, probably leading to such high prevalence rates.

In Central and Southern Germany (Region C), USUV is circulating since its first introduction into Germany in 2010/2011. In both years investigated, the percentage of USUV antibody-positive birds remained rather similar, in contrast to the other regions. In 2017, 3.86% and in 2018, 6.53% of the investigated birds were USUV seropositive. The high number of infected zoo birds in 2018 was remarkable, this indicates an ongoing local circulation of USUV in this region. In conclusion, regions where USUV was detected for the first time showed numerous and exceptionally high USUV antibody titers.

In the previous German monitoring studies of the years 2011–2016, the percentage of USUV antibody-positive birds were quite low, ranging between 0.89% (2011–2013) and 3.9% (in 2015) [5,6]. However, we calculated the yearly prevalence over all sampling sites and did not differentiate between the geographic regions. Additionally, USUV was not as widespread in Germany as in 2017 and 2018. Nevertheless, if we use the new data to calculate the seroprevalence rates for the whole country of Germany in 2017 (5.14%) and 2018 (8.44%), the prevalence rates are still higher than those in the six previous years.

As already discussed in the previous monitoring studies, the formation of a so-called herd immunity, with a seroconversion of over 50% as found in Austria, has not been seen in the wild birds in Germany between 2017 and 2018 [73]. However, in areas of extensive USUV circulation, like the region of Leipzig, a quite high seroprevalence was detected. Bosch et al. proposed that even after the formation of a herd immunity, due to the short lifespan of Eurasian Blackbirds, after several years, new generations can become infected again [74].

In a few birds of this study, the differentiation between WNV and USUV seropositive samples was not possible, as the neutralizing antibody titers against both viruses did not vary or differed only 1- to 1.5-fold (Table 7). As the ND_50_ values for both WNV and USUV were quite high in one Eurasian Blackbird (ND_50_ WNV 1:160 and ND_50_ USUV 1:240) and one House Sparrow (*Passer domesticus*) (ND_50_ WNV 1:60 and ND_50_ USUV 1:80), a co-infection with both viruses seems possible for these birds. A similar serological phenomenon was seen in the Zoo Halle, where one Snowy Owl (*Bubo scandiacus*) also had high neutralizing antibody titers against both viruses [75]. In the same zoo, WNV-specific RNA was detected in Germany for the first time (in 2018) in a Great Grey Owl. Nevertheless, another Great Grey Owl kept in the same aviary was tested USUV-RNA-positive [75]. This shows that in the same region, mosquitoes infected with either WNV or USUV are circulating, enabling a possible co-infection in birds.

Taken together, USUV has spread massively throughout Germany during the past two years, affecting many different bird species. Phylogenetic investigations revealed a spread, especially of the USUV lineages Africa 3 and Europe 3 towards Northern Germany. Furthermore, the lineage Europe 2 was detected for the first time in Germany. Neutralizing antibodies against USUV were predominantly detected among the resident bird population, with quite high seroprevalence rates and antibody titers in Northern Germany, where USUV emerged for the first time in 2018, but also in Eastern Germany.

Despite the first detection of WNV-RNA in several deceased birds in Eastern/Central Germany in 2018, we were not able to detect WNV in our live bird sample panel to date. However, we have seen the first indications that WNV is circulating in Germany. Especially in Eastern Germany, high neutralizing antibody titers have been detected among resident and short-distance migratory birds, indicating a recent virus contact.

## 5. Conclusions

It can be assumed that the extraordinarily high temperatures recorded for Germany in 2018 led to favorable conditions for mosquito reproduction and additionally, to a faster virus replication of the mosquito-borne flaviviruses WNV and USUV. Consequently, USUV was able to spread throughout the country and WNV was detected for the first time in Germany. Integrated surveillance of birds and mosquitoes and the cooperation between human and animal health are essential in detecting whether or not WNV, similarly to USUV, will establish in Germany and spread throughout the whole country. Therefore, the continuation of the systematic monitoring system for wild birds is important and necessary for Germany.

## Figures and Tables

**Table 1:** Total number of wild bird blood samples investigated between 2017 and 2018 in Germany, divided into bird orders.

Order	Year 2017	Year 2018	Total
Passeriformes	291	219	510
Accipitriformes/Falconiformes	208	236	444
Strigiformes	66	79	145
Anseriformes	26	49	75
Columbiformes	115	166	281
Apodiformes	26	8	34
Charadriiformes	25	49	74
Ciconiiformes/Pelicaniformes	13	20	33
Gruiformes	5	8	13
Piciformes	47	34	81
Suliformes	3	0	3
Galliformes	2	0	2
Podicipediformes	3	2	5
Coraciiformes	5	0	5
Procellariiformes	0	1	1
Cuculiformes	0	1	1
Psittaciformes	0	2	2
**Total**	**835**	**874**	**1709**

**Table 2:** Results of quantitative real-time polymerase chain reactions (qRT-PCR) in wild and zoo bird samples of the first sample panel in 2017 and 2018. Positive samples are highlighted in red.

Order	Common Name	Scientific Name	Migration Pattern	Housing	WNV qRT-PCR no.pos.	USUV qRT-PCR no.pos.	Federal State
**(a) qRT-PCR results in 2017**							
Passeriformes	Eurasian Blackbird	*Turdus merula*	R, P	wild	0	28	SN, ST, BE, HE
	Common Starling	*Sturnus vulgaris*	R, P, S	wild	0	1	HE
	Carrion Crow	*Corvus corone*	S	wild	0	1	NRW
	Western Jackdaw	*Coloeus monedula*	R, P	wild	0	1	NRW
Piciformes	Great Spottet Woodpecker	*Dendrocopos major*	R, P, (S)	wild	0	1	LS
**in Total in 2017**					0/826	32/826	
**(b) qRT-PCR results in 2018**							
Passeriformes	Eurasian Blackbird	*Turdus merula*	R, P	wild	0	13	SN, LS, HE
	House Sparrow	*Passer domesticus*	R	wild	0	1	BW
	Eurasian Bullfinch	*Pyrrhula pyrrhula*	/	captive	0	2	SN, SH
	Domestic Canary	*Serinus canaria forma domestica*	/	captive	0	1	NRW
	Grey-Headed Bullfinch	*Pyrrhula erythaca*	/	captive	0	1	LS
	Song Thrush	*Turdus philomelos*	R, S	wild	0	2	SN
	Common Starling	*Sturnus vulgaris*	R, P, S	wild	0	1	SN
Strigiformes	Great Grey Owl	*Strix nebulosa*	/	captive	0	2	BW, HE
	Little Owl	*Athene noctua*	R	wild	0	1	HE
Falconiformes	European Kestrel	*Falco tinnunculus*	R, P, S	wild	0	1	HE
**Total**					0/781	25/781	

R = resident species, P = partial migrant, S = short distance migrant, / = not applicable (captive birds), SN = Saxony, ST = Saxony-Anhalt, BE = Berlin, HE = Hesse, NRW = North Rhine-Westphalia, LS = Lower Saxony, SH = Schleswig-Holstein, BW = Baden-Wurttemberg.

**Table 3:** Dead birds of the second sample panel tested positive for Usutu virus (USUV) infection by qRT-PCR in 2017.

Order	Common Name	Scientific Name	Migration Pattern	Housing	USUV RNA Positive Birds
Passeriformes	Eurasian Blackbird	*Turdus merula*	R, P	wild	94
	Song Trush	*Turdus philomelos*	R, L	wild	1
	Golden-breasted Starling	*Cosmopsarus regius*	/	captive	1
Strigiformes	Great Grey Owl	*Strix nebulosa*	/	captive	2
	Northern Hawk-owl	*Surnia ulula*	/	captive	2
**in Total in 2017**					**100**

R = resident species, P = partial migrant, S = short distance migrant, L = long distance migrant, / = not applicable (captive birds).

**Table 4:** WNV- and USUV-positive neutralization assay results from wild bird serum samples Region A—Northern Germany.

Sample Collector	Order	Common Name	Scientific Name	Migration Pattern	No. Samples Tested	WNV Pos. (ND_50_)	USUV Pos. (ND_50_)
**(a) WNV and USUV positive neutralization assay results (positives highlighted in red and bold) from wild bird serum in 2017. Cross-reacting antibody titers are also displayed in black**							
①	Piciformes	Great Spottet Woodpecker	*Dendrocopos major*	R, P, (S)	7	0	**1 (10)**
②	Accipitriformes	Common Buzzard	*Buteo buteo*	R, P, S	11	**1 (10)**	0
②	Columbiformes	Common Wood Pigeon	*Columba palumbus*	R, P, S	29	**2 (10), 1 (15)**	0
		Feral Pigeon	*Columba livia* f. *domestica*	R, (P)	14	1 (10)	**1 (20)**
②	Charadriiformes	Eurasian Woodcock	*Scolopax rusticola*	R, S	**3**	1 (10)	**1 (20)**
②	Suliformes	Great Cormorant	*Phalacrocorax carbo*	R, S	1	**1 (10)**	0
**Total**					**171**	**5**	**3**
**Seroprevalence**						**2.92%**	**1.75%**
**(b) WNV- and USUV-positive neutralization assay results (positives highlighted in red and bold) from wild bird serum in 2018. Cross-reacting antibody titers are also displayed in black**							
①	Passeriformes	Eurasian Blackbird	*Turdus merula*	R, P	16	1 (10), 1 (20), 1 (60)	**2 (10), 1 (15), 1 (40), 1 (60),** **1 (240), 1 (320)**
①		Carrion Crow	*Corvus corone*	R, P	14	**1 (10)**	0
①		Spotted Flycatcher	*Muscicapa striata*	L	2	**1 (10)**	0
①	Accipitriformes	Northern Goshawk	*Accipiter gentilis*	R, P	1	0	**1 (10)**
①		Common Buzzard	*Buteo buteo*	R, P, S	6	0	**1 (40)**
①	Anseriformes	Northern Mallard Duck	*Anas platyrhynchos*	R, P, S	5	1 (20)	**1 (10), 1 (480)**
①	Ciconiiformes/ Pelicaniformes	Northern Gannet	*Morus bassanus*	S, L	6	**1 (10)**	0
②	Passeriformes	Eurasian Blackbird	*Turdus merula*	R, P	9	1 (120), 1 (240)	**1 (10), 1 (240), 1 (1280)**
②		Eurasian Jay	*Garrulus glandarius*	R, P	3	**1 (15)**, 1 (40)	**1 (1280)**
②		Common Magpie	*Pica pica*	R	5	**1 (15)**	0
②		Common Starling	*Sturnus vulgaris*	R, P, S	2	1 (40)	**1 (240)**
②	Accipitriformes	Common Buzzard	*Buteo buteo*	R, P, S	11	**2 (15)**	0
②	Columbiformes	Common Wood Pigeon	*Columba palumbus*	R, P, S	48	1 (10), 1 (15), 1 (20), 1 (40)	**1 (40), 3 (80)**
②	Apodiformes	Common Swift	*Apus apus*	L	1	0	**1 (10)**
③	Strigiformes	Eurasian Eagle Owl	*Bubo bubo*	zoo bird	5	0	**1 (30), 1 (20), 1 (15)**
③		Snowy Owl	*Bubo scandiacus*	zoo bird	2	0	**1 (640)**
③		Ural Owl	*Strix uralensis*	zoo bird	2	1 (30)	**1 (320)**
③		Northern Long-eared Owl	*Asio otus*	zoo bird	3	1 (20)	**1 (80)**
③		Eurasian Tawny Owl	*Strix aluco*	zoo bird	2	1 (10), 1 (60)	**1 (160), 1 (2560)**
**Total**					**304**	**7**	**29**
**Seroprevalence**						**2.30%**	**9.54%**

R = resident species, P = partial migrant, S = short distance migrant, L = long distance migrant, (For details regarding the sample collectors see Section 2.1 and for ND_50_ Section 2.4).

**Table 5:** WNV- and USUV-positive neutralization assay results from wild bird serum samples Region B—Eastern Germany.

Sample Collector	Order	Common Name	Scientific Name	Migration Pattern	No. Samples Tested	WNV Pos. (ND_50_)	USUV Pos. (ND_50_)
**(a) WNV- and USUV-positive neutralization assay results (positives highlighted in red and bold) from wild bird serum in 2017. Cross-reacting antibody titers are also displayed in black**							
④	Charadriiformes	Eurasian Woodcock	*Scolopax rusticola*	R, S	7	2 (10), 1 (20)	**1 (10), 1 (25), 1 (30), 1 (40)**
④	Ciconiiformes/Pelicaniformes	Grey Heron	*Ardea cinerea*	R, P, S	1	0	**1 (10)**
⑤	Passeriformes	Eurasian Blackbird	*Turdus merula*	R, P	11	1 (10), 1 (15), 1 (20)	**1 (20), 1 (40), 1 (50), 1 (80)**
⑤	Falconiformes	European Kestrel	*Falco tinnunculus*	R, P, S	5	**1 (20), 1 (80)**	0
⑤	Columbiformes	Common Wood Pigeon	*Columba palumbus*	R, P, S	9	3 (10), 1 (60)	**2 (20), 1 (30), 1 (80),** **1 (320), 1 (480)**
⑤	Ciconiiformes/Pelicaniformes	White Stork	*Ciconia ciconia*	L	1	1 (20)	**1 (40)**
**Total**					**76**	**2**	**16**
**Seroprevalence**						**2.63%**	**21.05%**
**(b) WNV- and USUV-positive neutralization assay results (positives highlighted in red and bold) from wild bird serum in 2018. Cross-reacting antibody titers are also displayed in black**							
④	Passeriformes	Common Magpie	*Pica pica*	R	1	0	**1 (10)**
④	Accipitriformes	Northern Goshawk	*Accipiter gentilis*	R, P	2	**1 (320)**	0
④		White-tailed Eagle	*Haliaeetus albicilla*	R, P	5	**1 (240)**	0
④		Common Buzzard	*Buteo buteo*	R, P, S	7	**1 (20)**	1 (10)
④	Falconiformes	European Kestrel	*Falco tinnunculus*	R, P, S	1	**1 (160)**	1 (40)
④	Strigiformes	Northern Long-eared Owl	*Asio otus*	R, P, S	2	1 (10)	**1 (60)**
④	Columbiformes	Common Wood Pigeon	*Columba palumbus*	R, P, S	2	0	**1 (10)**
④	Anseriformes	Northern Mallard Duck	*Anas platyrhynchos*	R, P, S	2	0	**1 (10)**
④	Apodiformes	Common Swift	*Apus apus*	L	1	1 (10)	**1 (30)**
④	Charadriiformes	Eurasian Woodcock	*Scolopax rusticola*	R, S	12	0	**1 (10)**
⑤	Passeriformes	Eurasian Blackbird	*Turdus merula*	R, P	3	0	**1 (60)**
⑤		Carrion Crow	*Corvus corone*	P, S	5	**1 (240)**	1 (40)
⑤	Accipitriformes	Common Buzzard	*Buteo buteo*	R, P, S	2	0	**1 (60), 1 (10)**
⑤	Columbiformes	Common Wood Pigeon	*Columba palumbus*	R, P, S	7	**1 (80)**, 1 (60), 1 (30)	**1 (320), 1 (80), 1 (30)**, 1 (10)
**Total**					**92**	**6**	**12**
**Seropevalence**						**6.52%**	**13.04%**

R = resident species, P = partial migrant, S = short distance migrant, L = long distance migrant, (For details regarding the sample collectors see Section 2.1 and for ND_50_ Section 2.4).

**Table 6:** WNV- and USUV-positive neutralization assay results from wild bird serum samples Region C—Central and Southern Germany.

Sample Collector	Order	Common Name	Scientific Name	Migration Pattern	No. Samples Tested	WNV Pos. (ND_50_)	USUV Pos. (ND_50_)
**(a) WNV- and USUV-positive neutralization assay results (positives highlighted in red and bold) from wild bird serum in 2017. Cross-reacting antibody titers are also displayed in black**							
⑤	Passeriformes	Eurasian Blackbird	*Turdus merula*	R, P	4	1 (75), 1 (190)	** 1 (160), 1 (3840)**
⑥	Passeriformes	Eurasian Blackbird	*Turdus merula*	R, P	23	**1 (15)**	0
⑥		Eurasian Blue Tit	*Cyanistes caeruleus*	R	2	**1 (10)**	0
⑥		Thrush	*Turdus* sp.	R, S	10	**1 (10)**	**1 (15)**
⑥		Common Starling	*Sturnus vulgaris*	R, P, S	5	**1 (10)**	0
⑥	Accipitriformes	Long-legged Buzzard	*Buteo rufinus*	zoo bird	2	0	**1 (10)**
⑥		Common Buzzard	*Buteo buteo*	R, P, S	24	**2 (10)**, 1 (10)	**1 (60), 1 (15)**
⑥		African Sea Eagle	*Haliaeetus vocifer*	zoo bird	3	**1 (30)**	0
⑥		Black Kite	*Milvus migrans*	L	2	**1 (10)**	0
⑥	Falconiformes	European Kestrel	*Falco tinnunculus*	R, P, S	21	**2 (15), 1 (30)**	0
⑥	Columbiformes	Common Wood Pigeon	*Columba palumbus*	R, P, S	16	0	**2 (10), 1 (60)**
⑥	Apodiformes	Common Swift	*Apus apus*	L	10	**1 (10)**	**1 (15)**
⑥	Ciconiiformes/Pelicaniformes	Grey Heron	*Ardea cinerea*	R, P, S	7	1 (15)	**1 (40)**
⑥		Stork sp.	*Circonia* sp.	L	1	**1 (10)**	0
⑦	Gruiformes	Eurasian coot	*Fulica atra*	P, S	1	1 (15)	**1 (30)**
⑧	Accipitriformes	Common Buzzard	*Buteo buteo*	R, P, S	13	**1 (20)**, 1 (10)	**2 (320), 1 (20), 1 (15), 1 (10)**
⑧		Northern Goshawk	*Accipiter gentilis*	R, P	2	0	**1 (15)**
⑧	Strigiformes	Eurasian Tawny Owl	*Strix aluco*	R	2	0	**1 (80)**
⑧	Gruiformes	Eurasian Coot	*Fulica atra*	P, S	1	**1 (10)**	0
**Total**					**492**	**15**	**19**
**Seroprevalence**						**3.05%**	**3.86%**
**(b) WNV- and USUV-positive neutralization assay results (positives highlighted in red and bold) from wild bird serum in 2018. Cross-reacting antibody titers are also displayed in black**							
⑥	Passeriformes	Eurasian Blackbird	*Turdus merula*	R, P, S	19	**2 (15)**, 1 (60), 1 (10)	**1 (320), 1 (30), 1 (20)**
⑥		Common Magpie	*Pica pica*	R	6	**1 (15)**	0
⑥		Carrion Crow	*Corvus corone*	P, S	9	**1 (40)**	0
⑥		European Robin	*Erithacus rubecula*	P	2	0	**1 (15)**
⑥	Accipitriformes/Falconiformes	Black-chested Buzzard-eagle	*Geranoaetus melanoleucus*	zoo bird	1	0	**1 (10)**
⑥		Griffon Vulture	*Gyps fulvus*	zoo bird	4	0	**1 (15)**
⑥		Northern Goshawk	*Accipiter gentilis*	R, P	12	0	**1 (10)**
⑥		Eastern Imperial Eagle	*Aquila heliaca*	zoo bird	1	0	**1 (15)**
⑥		Common Buzzard	*Buteo buteo*	R, P, S	25	0	**1 (15), 1 (20)**
⑥		Western Marsh Harrier	*Circus aeruginosus*	L	6	**1 (40), 1 (20)**	**1 (10)**
⑥		Southern Crested Caracara	*Caracara plancus*	zoo bird		**1 (10)**	0
⑥		Rüppell′s Vulture	*Gyps rueppelli*	zoo bird	1	1 (15)	**1 (50)**
⑥		Golden Eagle	*Aquila chrysaetos*	zoo bird	4	0	**1 (60)**
⑥		White-backed Vulture	*Gyps africanus*	zoo bird	1	**1 (20)**	0
⑥		Eurasian Hobby	*Falco subbuteo*	L	2	**1 (30)**	0
⑥		European Kestrel	*Falco tinnunculus*	R, P, S	14	**1 (20)**	0
⑥	Strigiformes	Northern Long-eared Owl	*Asio otus*	R, P, S	4	0	**1 (30)**
⑥	Columbiformes	Common Wood Pigeon	*Columba palumbus*	R, P, S	21	1 (40)	**1 (80)**
⑥		Feral Pigeon	*Columba livia* f. *domestica*	R, (P)	13	0	**2 (10), 1 (15)**
⑥	Anseriformes	Mute Swan	*Cygnus olor*	R, P, S	9	**1 (10)**	**1 (15), 1 (40)**
⑥		Egyptian Goose	*Alopochen aegyptiacus*	R (Neozoa)	2	0	**1 (10)**
⑥	Ciconiiformes/Pelicaniformes	Grey Heron	*Ardea cinerea*	R, P, S	8	0	**1 (10), 1 (40)**
⑥	Cuculiformes	Common Cuckoo	*Cuculus canorus*	L	1	**1 (20)**	0
⑥	Piciformes	Great Spottet Woodpecker	*Dendrocopos major*	R, P, (S)	6	**1 (10)**	0
⑦	Columbiformes	Feral Pigeon	*Columba livia* f. *domestica*	R, (P)	16	0	**1 (30)**
⑧	Accipitriformes	Common Buzzard	*Buteo buteo*	R, P, S	3	0	**1 (15)**
⑧	Falconiformes	European Kestrel	*Falco tinnunculus*	R, P, S	1	0	**1 (30)**
**Total**					**398**	**13**	**26**
**Seroprevalence**						**3.27%**	**6.53%**

R = resident species, P = partial migrant, S = short distance migrant, L = long distance migrant, (For details regarding the sample collectors see Section 2.1 and for ND_50_ Section 2.4).

**Table 7:** No differentiation between WNV and USUV by neutralization assay possible.

Sample Collector	Year	Common Name	Scientific Name	WNV pos. (ND_50_)	USUV pos. (ND_50_)
②	2017	Common Wood Pigeon	*Columba palumbus*	1 (10)	1 (15)
②	2017	Common Wood Pigeon	*Columba palumbus*	1 (10)	1 (15)
⑤	2017	Eurasian Blackbird	*Turdus merula*	1 (15)	1 (20)
⑤	2017	Eurasian Blackbird	*Turdus merula*	1 (160)	1 (240)
⑥	2017	Northern Goshawk	*Accipiter gentilis*	1 (15)	1 (10)
⑥	2017	European Kestrel	*Falco tinnunculus*	1 (15)	1 (15)
⑥	2017	Snowy owl	*Bubo scandiacus*	1 (10)	1 (10)
①	2018	Eurasian Blackbird	*Turdus merula*	1 (30)	1 (40)
⑥	2018	Eurasian Blackbird	*Turdus merula*	1 (30)	1 (30)
⑥	2018	Eurasian Blackbird	*Turdus merula*	1 (10)	1 (10)
⑥	2018	Eurasian Blackbird	*Turdus merula*	1 (20)	1, (15)
⑥	2018	House Sparrow	*Passer domesticus*	1 (60)	1 (80)
⑥	2018	Great Grey Owl	*Strix nebulosa*	1 (30)	1 (20)
⑥	2018	Stork sp.	*Circonia* sp.	1 (10)	1 (15)

## Supplementary Materials

The following are available online at https://www.mdpi.com/1999-4915/11/7/674/s1, Table S1: WNV and USUV neutralization assay results from wild bird blood samples between 2017 and 2018. Table S2: Detailed information on the origin of the phylogenetically analyzed wild and captive birds of the live and dead bird monitoring in 2017 and 2018.
