# Supplementary material for: Evidence for West Nile Virus and Usutu Virus Infections in Wild and Resident Birds in Germany, 2017 and 2018
Source: Viruses. 2019 Jul 23;11(7):674. doi: 10.3390/v11070674 (PMC6669720; doi:10.3390/v11070674)
Supplement: Supplementary file 1 [file viruses-11-00674-s001.zip › Table S1.WNV and USUV neutralization assay results.docx]

**Table S1.** WNV and USUV neutralization assay results from wild bird blood samples between 2017 and 2018. Positive samples are highlighted in red and bold, neutralization titres in brackets. Cross reacting antibody titers are also displayed in black.

| Order | Common Name | Scientific Name | Migration Pattern | No. Samples Tested | WNV Pos. (ND_50_) | USUV Pos. (ND_50_) |
| --- | --- | --- | --- | --- | --- | --- |
| Accipitriformes | Long-legged Buzzard | *Buteo rufinus* | zoo bird | 4 | 0 | **1 (10)** |
|  | Black-chested Buzzard-eagle | *Geranoaetus melanoleucus* | zoo bird | 2 | 0 | **1 (10)** |
|  | Bearded Vulture | *Gypaetus barbatus* | zoo bird | 1 | 0 | 0 |
|  | White-tailed Eagle | *Haliaeetus albicilla* | R, P | 20 | **1 (240)** | 0 |
|  | Osprey | *Pandion haliaetus* | L | 1 | 0 | 0 |
|  | Griffon Vulture | *Gyps fulvus* | zoo bird | 9 | 0 | **1 (15)** |
|  | Northern Goshawk | *Accipiter gentilis* | R, P | 26 | **1 (320)**, 1 (15) | **2 (10), 1 (15),** 1 (10) |
|  | Bonelli's Eagle | *Hieraaetus fasciatus* | zoo bird | 1 | 0 | 0 |
|  | Eastern Imperial Eagle | *Aquila heliaca* | zoo bird | 2 | 0 | **1 (15)** |
|  | King Vulture | *Sarcoramphus papa* | zoo bird | 2 | 0 | 0 |
|  | Ferruginous Hawk | *Buteo regalis* | zoo bird | 4 | 0 | 0 |
|  | Common Buzzard | *Buteo buteo* | R, P, S | 126 | **3 (10), 2 (15), 2 (20)**, 2 (10) | **2 (10), 4 (15), 2 (20), 1 (40),**  **2 (60), 2 (320),** 1 (10) |
|  | Cinereous Vulture | *Aegypius monachus* | zoo bird | 4 | 0 | 0 |
|  | Steller's Sea Eagle | *Haliaeetus pelagicus* | zoo bird | 3 | 0 | 0 |
|  | Western Marsh Harrier | *Circus aeruginosus* | L | 9 | **1 (40), 1 (20)** | **1 (10)** |
|  | Red Kite | *Milvus milvus* | (R), S | 6 | 0 | 0 |
|  | African Sea Eagle | *Haliaeetus vocifer* | zoo bird | 5 | **1 (30)** | 0 |
|  | Black Kite | *Milvus migrans* | L | 3 | **1 (10)** | 0 |
|  | Eurasian Sparrowhawk | *Accipiter nisus* | R, P, S | 29 | 0 | 0 |
|  | Rüppell's Vulture | *Gyps rueppelli* | zoo bird | 2 | 1 (15) | **1 (50)** |
|  | Golden Eagle | *Aquila chrysaetos* | zoo bird | 9 | 0 | **1 (60)** |
|  | Golden Eagle X Steppe Eagle Hybrid |  | zoo bird | 1 | 0 | 0 |
|  | Steppe Eagle | *Aquila nipalensis* | zoo bird | 8 | 0 | 0 |
|  | Bald Eagle | *Haliaeetus leucocephalus* | zoo bird | 12 | 0 | 0 |
|  | White-backed Vulture | *Gyps africanus* | zoo bird | 1 | **1 (20)** | 0 |
|  | European Honey Buzzard | *Pernis apivorus* | L | 2 | 0 | 0 |
|  | Harris-Hawk | *Parabuteo unicinctus* | zoo bird | 16 | 0 | 0 |
| Anseriformes | Common Shelduck | *Tadorna tadorna* | S | 2 | 0 | 0 |
|  | Graylag Goose | *Anser anser* | R, P, S | 7 | 0 | 0 |
|  | Mute Swan | *Cygnus olor* | R, P, S | 32 | **1 (10)**, 1* | **1 (15), 1 (40)** |
|  | Canada Goose | *Branta canadensis* | zoo bird, R (neozoa) | 4 | 0 | 0 |
|  | Indian Runners | *Anas platyrhynchos* f. *domestica* | zoo bird | 1 | 0 | 0 |
|  | Egyptian Goose | *Alopochen aegyptiacus* | R (neozoa) | 6 | 0 | **1 (10)** |
|  | Barnacle Goose | *Branta leucopsis* | L | 1 | 0 | 0 |
|  | Northern Mallard Duck | *Anas platyrhynchos* | R, P, S | 22 | 1 (20), 1* | **2 (10), 1 (480)** |
| Apodiformes | Common Swift | *Apus apus* | L | 32 | **1 (10)**, 1 (10), 5* | **1 (10), 1 (15), 1 (30)** |
| Charadriiformes | Eurasian Oystercatcher | *Haematopus ostralegus* | S | 4 | 0 | 0 |
|  | Common Curlew | *Numenius arquata* | S | 1 | 0 | 0 |
|  | Lesser Black-backed Gull | *Larus fuscus* | P, S, L | 3 | 0 | 0 |
|  | Northern Lapwing | *Vanellus vanellus* | S | 1 | 0 | 0 |
|  | Red Knot | *Calidris canutus* | L | 1 | 0 | 0 |
|  | Black-headed Gull | *Larus ridibundus* | R, P, S | 15 | 1 * | 0 |
|  | Gull | *Laridae (Fam.)* | R, S, L | 1 | 0 | 0 |
|  | European Herring Gull | *Larus argentatus* | R, S | 9 | 0 | 0 |
|  | Common Gull | *Larus canus* | R, S | 1 | 0 | 0 |
|  | Common Guillemot | *Uria aalge* | R, S | 4 | 0 | 0 |
|  | Eurasian Woodcock | *Scolopax rusticola* | R, S | 31 | 3 (10), 1 (20) | **2 (10), 1 (20), 1 (25), 1 (30),**  **1 (40)** |
| Ciconiiformes/ Pelicaniformes | Grey Heron | *Ardea cinerea* | R, P, S | 18 | 1 (15) | **2 (10), 2 (40)** |
|  | Stork sp. | *Ciconia* sp. | L | 3 | **1 (10),** 1 (10) | 1 (15) |
|  | White Stork | *Ciconia ciconia* | L | 3 | 1 (20) | **1 (40)** |
|  | Northern Gannet | *Morus bassanus* | S, L | 6 | **1 (10)** | 0 |
| Columbiformes | Common Wood Pigeon | *Columba palumbus* | R, P, S | 152 | **2 (10), 1 (15), 1 (80),** 6 (10), 1 (15), 1 (20), 1 (30), 2 (40), 2 (60), 1* | **3 (10), 2 (20), 2 (30), 1 (40),**  **1 (60), 6 (80), 2 (320), 1 (480)**, 1 (10), 2 (15) |
|  | Feral Pigeon | *Columba livia* f. *domestica* | R, (P) | 100 | 1 (10), 1 # | **2 (10), 1 (15), 1 (20), 1 (30)**, 1 * |
|  | Pigeon | *Columba* sp. | R, (P) | 4 | 0 | 0 |
|  | Eurasian Collared Dove | *Streptopelia decaocto* | R, (P) | 15 | 0 | 0 |
| Coraciiformes | Common Kingfisher | *Alcedo atthis* | R, S | 4 | 0, 1* | 0 |
| Cuculiformes | Common Cuckoo | *Cuculus canorus* | L | 1 | **1 (20)** | 0 |
| Falconiformes | Eurasian Hobby | *Falco subbuteo* | L | 3 | **1 (30)** | 0 |
|  | Striated Caracara | *Phalcoboenus australis* | zoo bird | 2 | 0 | 0 |
|  | Gyrfalcon | *Falco rusticolus* | zoo bird | 5 | 0 | 0 |
|  | Gyrfalcon X Lanner Falcon Hybrid |  | zoo bird | 1 | 0 | 0 |
|  | Lanner Falcon | *Falco biarmicus* | zoo bird | 2 | 0 | 0 |
|  | Laggar Falcon | *Falco jugger* | zoo bird | 1 | 0 | 0 |
|  | Merlin | *Falco columbarius* | zoo bird | 1 | 0 | 0 |
|  | Saker Falcon | *Falco cherrug* | zoo bird | 6 | 0 | 0 |
|  | Southern Crested Caracara | *Caracara plancus* | zoo bird | 3 | **1 (10)** | 0 |
|  | European Kestrel | *Falco tinnunculus* | R, P, S | 60 | **2 (15), 2 (20), 1 (30), 1 (80), 1 (160)**, 1 (15) | **1 (30)**, 1 (15), 1 (40) |
|  | Peregrine Falcon | *Falco peregrinus* | R, P | 24 | 0 | 0 |
| Galliformes | Common Pheasant | *Phasianus colchicus* | R | 2 | 0 | 0 |
| Gruiformes | Eurasian Coot | *Fulica atra* | P, S | 2 | **1 (10)**, 1 (15) | **1 (30)** |
|  | Common Crane | *Grus grus* | S, L | 1 | 0 | 0 |
|  | Common Moorhen | *Gallinula chloropus* | R, P, S | 6 | 0 | 0 |
| Passeriformes | Eurasian Blackbird | *Turdus merula* | R, P | 118 | **3 (15)**, 4 (10), 2 (15), 3 (20), 2 (30), 2 (60), 1 (75), 1 (120), 1 (160), 1 (190), 1 (240), 4* | **3 (10), 1 (15), 2 (20), 1 (30),**  **2 (40), 1 (50), 2 (60), 1 (80),**  **1 (160), 2 (240), 2 (320), 1 (1280), 1 (3840),** 1 (10), 1, (15), 1 (20), 1 (30), 1 (40), 1 (240), 3* |
|  | Eurasian Blue Tit | *Cyanistes caeruleus* | R | 5 | **1 (10)** | 0, 1* |
|  | Common Linnet | *Linaria cannabina* | R, S | 2 | 0 | 0 |
|  | Common Chaffinch | *Fringilla coelebs* | R, P | 6 | 0, 1* | 0 |
|  | Western Jackdaw | *Coloeus monedula* | S | 10 | 0 | 0 |
|  | Eurasian Bullfinch | *Pyrrhula pyrrhula* | R, P | 1 | 0 | 1* |
|  | Thrush | *Turdus* sp. | S, L | 13 | **1 (10)** | **1 (15)** |
|  | Eurasian Jay | *Garrulus glandarius* | R, P | 18 | **1 (15)**, 1 (40) | **1 (1280)** |
|  | Common Magpie | *Pica pica* | R | 32 | **2 (15)**, 1# | **1 (10)** |
|  | Eurasian Siskin | *Carduelis spinus* | S | 1 | 0 | 0 |
|  | Garden Warbler | *Sylvia borin* | L | 1 | 0 | 0 |
|  | Common Redstart | *Phoenicurus phoenicurus* | L | 1 | 0 | 0 |
|  | Yellowhammer | *Emberiza citrinella* | R, P, S | 1 | 0 | 0 |
|  | Typical Warbler | *Sylvia* sp. | L | 1 | 0 | 0 |
|  | Spotted Flycatcher | *Muscicapa striata* | L | 2 | **1 (10)** | 0 |
|  | European Greenfinch | *Carduelis chloris* | S | 8 | 0 | 0 |
|  | Black Redstart | *Phoenicurus ochruros* | P, S | 4 | 0 | 0 |
|  | House Sparrow | *Passer domesticus* | R | 20 | 1 (60), 3 * | 1 (80) |
|  | Grosbeak | *Coccothraustes coccothraustes* | S | 5 | 0 | 0 |
|  | Eurasian Nuthatch | *Sitta europaea* | R | 2 | 0 | 0 |
|  | Great Tit | *Parus major* | R, (P) | 8 | 0 | 0 |
|  | Common House Martin | *Delichon urbicum* | L | 9 | 0 | 0 |
|  | Eurasian Blackcap | *Sylvia atricapilla* | S, L | 4 | 0 | 0 |
|  | Carrion Crow | *Corvus corone* | P, S | 88 | **1 (10), 1 (40), 1 (240)**, 1*, 1# | 1 (40) |
|  | Barn Swallow | *Hirundo rustica* | L | 9 | 0, 1* | 0 |
|  | European Robin | *Erithacus rubecula* | P | 9 | 0, 1* | **1 (15)** |
|  | Rook | *Corvus frugilegus* | R, P, S | 1 | 0 | 0 |
|  | Long-tailed Tit | *Aegithalos caudatus* | R, S | 1 | 0 | 0 |
|  | Song Thrush | *Turdus philomelos* | R, S | 9 | 0 | 0 |
|  | Eurasian Tree Sparrow | *Passer montanus* | R | 2 | 0 | 0 |
|  | Common Starling | *Sturnus vulgaris* | R, P, S | 9 | **1 (10)**, 1 (40) | **1 (240)** |
|  | Northern Wheatear | *Oenanthe oenanthe* | L | 1 | 0 | 0 |
|  | European Goldfinch | *Carduelis carduelis* | R, P | 1 | 0 | 0 |
|  | Fieldfare | *Turdus pilaris* | S | 7 | 0 | 0 |
|  | Goldcrest | *Regulus regulus* | R, S, L | 1 | 0 | 0 |
| Piciformes | Great Spottet Woodpecker | *Dendrocopos major* | R, P, (S) | 35 | **1 (10)**, 1* | **1 (10)** |
|  | Eurasian Green Woodpecker | *Picus viridis* | R, (P) | 29 | 0, 1* | 0 |
|  | Lesser Spotted Woodpecker | *Dryobates minor* | R, S | 2 | 0 | 0 |
|  | Middle Spotted Woodpecker | *Dendrocopos medius* | R | 4 | 0, 1* | 0 |
|  | Black Woodpecker | *Dryocopus martius* | R, P | 1 | 0 | 0 |
| Podicipediformes | Great Crested Grebe | *Podiceps cristatus* | R | 4 | 0 | 0 |
| Procellariiformes | Northern Fulmar | *Fulmarus glacialis* | R | 1 | 0 | 0 |
| Psittaciformes | Turquoise-fronted Amazon | *Amazona aestiva* | zoo bird | 1 | 0 | 0 |
|  | Palm Cockatoo | *Probosciger aterrimus* | zoo bird | 1 | 0 | 0 |
| Strigiformes | Great Horned Owl | *Bubo virginianus* | zoo bird | 3 | 0 | 0 |
|  | Great Grey Owl | *Strix nebulosa* | zoo bird | 9 | 1 (30) | 1 (20) |
|  | Owl | *Strigidae (Fam.)* | R, P, S, L | 1 | 0 | 0 |
|  | Spotted Eagle-owl | *Bubo africanus* | zoo bird | 5 | 0 | 0 |
|  | Ural Owl | *Strix uralensis* | zoo bird | 6 | 1 (30) | **1 (320)** |
|  | Boreal Owl | *Aegolius funereus* | R, P | 2 | 0 | 0 |
|  | Common Barn Owl | *Tyto alba* | R, P | 19 | 0 | 0 |
|  | Snowy Owl | *Bubo scandiacus* | zoo bird | 13 | 1 (10) | **1 (640)**, 1 (10) |
|  | Northern Hawk Owl | *Surnia ulula* | zoo bird | 1 | 0 | 0 |
|  | Little Owl | *Athene noctua* | R | 4 | 0 | 0 |
|  | Short-eared Owl | *Asio flammeus* | L | 1 | 0 | 0 |
|  | Eurasian Eagle Owl | *Bubo bubo* | R | 27 | 0 | **1 (30), 1 (20), 1 (15)** |
|  | Eurasian Tawny Owl | *Strix aluco* | R | 14 | 1 (10), 1 (60) | **1 (80), 1 (160), 1 (2560)** |
|  | Northern Long-eared Owl | *Asio otus* | R, P, S | 23 | 1 (10), 1 (20) | **1 (30), 1 (60), 1 (80)** |
|  | Eurasian Scops Owl | *Otus scops* | S, L | 3 | 0, 1* | 0 |
| Suliformes | Great Cormorant | *Phalacrocorax carbo* | R, S | 3 | **1 (10)** | 0 |

* Not done because insufficient serum volume for both tests # Not analyzable because sample is cytotoxic or coverings on the cells. R = resident species, P = partial migrant, S = short distance migrant, L = long distance migrant, (For details regarding the sample collectors see section 2.1 and for ND50 section 2.4.)
