# Supplementary material for: Evidence for West Nile Virus and Usutu Virus Infections in Wild and Resident Birds in Germany, 2017 and 2018
Source: Viruses. 2019 Jul 23;11(7):674. doi: 10.3390/v11070674 (PMC6669720; doi:10.3390/v11070674)
Supplement: Supplementary file 1 [file viruses-11-00674-s001.zip › Table S2. Detailed information.docx]

**Table S2.** Detailed information on the origin of the phylogenetically analyzed wild and captive birds of the live and dead bird monitoring in 2017 and 2018.

| **Year** | **GenBank Accesion No.** | **Common name** | **Scientific name** | **USUV Lineage** | **Origin (City/ Federal State)** |
| --- | --- | --- | --- | --- | --- |
| 2017 | MK956044 | Eurasian Blackbird | *Turdus merula* | **Europe 3** | Leipzig (SN) |
| 2017 | MK956045 | Eurasian Blackbird | *Turdus merula* | **Europe 3** | Leipzig (SN) |
| 2017 | MK956046 | Eurasian Blackbird | *Turdus merula* | **Africa 3** | Leipzig (SN) |
| 2017 | MK956047 | Eurasian Blackbird | *Turdus merula* | **Africa 3** | Leipzig (SN) |
| 2017 | MK956048 | Eurasian Blackbird | *Turdus merula* | **Africa 3** | Leipzig (SN) |
| 2017 | MK956049 | Eurasian Blackbird | *Turdus merula* | **Africa 3** | Leipzig (SN) |
| 2017 | MK956050 | Eurasian Blackbird | *Turdus merula* | **Africa 2** | Leipzig (SN) |
| 2017 | MK956051 | Eurasian Blackbird | *Turdus merula* | **Europe 3** | Leipzig (SN) |
| 2017 | MK956052 | Eurasian Blackbird | *Turdus merula* | **Africa 2** | Leipzig (SN) |
| 2017 | MK956054 | Eurasian Blackbird | *Turdus merula* | **Africa 2** | Berlin (BE) |
| 2017 | MK956053 | Eurasian Blackbird | *Turdus merula* | **Europe 3** | Karben (HE) |
| 2017 | MK956013 | Eurasian Blackbird | *Turdus merula* | **Africa 3** | Brüggen (NRW) |
| 2017 | MK956015 | Northern hawk-owl | *Surnia ulula* | **Europe 3** | Dresden (SN) |
| 2017 | MK956056 | Eurasian Blackbird | *Turdus merula* | **Africa 3** | Doberschütz (SN) |
| 2018 | MK956038 | Eurasian Blackbird | *Turdus merula* | **Africa 3** | Theene (LS) |
| 2018 | MK956028 | Eurasian Blackbird | *Turdus merula* | **Africa 2** | Leipzig (SN) |
| 2018 | M 669 (sequence identical to MK956046) | Eurasian Blackbird | *Turdus merula* | **Africa 3** | Leipzig (SN) |
| 2018 | MK956029 | Eurasian Blackbird | *Turdus merula* | **Africa 3** | Leipzig (SN) |
| 2018 | MK956030 | Eurasian Blackbird | *Turdus merula* | **Africa 3** | Leipzig (SN) |
| 2018 | MK956034 | Eurasian Bullfinch | *Pyrrhula pyrrhula* | **Africa 3** | Pinneberg (SH) |
| 2018 | MK956033 | House Sparrow | *Passer domesticus* | **Europe 3** | Freiburg im Breisgau (BW) |
| 2018 | MK956032 | Grey-Headed Bullfinch | *Pyrrhula erythaca* | **Europe 3** | Dinklage (LS) |
| 2018 | MK956031 | Eurasian Bullfinch | *Pyrrhula pyrrhula* | **Europe 3** | Groß Düben (SN) |
| 2018 | MK956040 | Song Thrush | *Turdus philomelos* | **Europe 2** | Leipzig (SN) |
| 2018 | MK956036 | Song Thrush | *Turdus philomelos* | **Europe 3** | Leipzig (SN) |
| 2018 | MK956037 | Common Starling | *Sturnus vulgaris* | **Europe 3** | Leipzig (SN) |
| 2018 | MK956035 | Great Grey Owl | *Strix nebulosa* | **Europe 3** | Stutensee (BW) |
| 2018 | MK956039 | Great Grey Owl | *Strix nebulosa* | **Europe 3** | Ranstadt (HE) |
| 2018 | MK956018 | Eurasian Blackbird | *Turdus merula* | **Europe 3** | Saarbrücken (SL) |
| 2018 | MK956010 | Great Grey Owl | *Strix nebulosa* | **Europe 3** | Nuremberg (BY) |
| 2018 | MK956017 | Great Grey Owl | *Strix nebulosa* | **Europe 3** | Nuremberg (BY) |
| 2018 | MK956020 | Eurasian Blackbird | *Turdus merula* | **Europe 3** | Munich (BY) |
| 2018 | MK956021 | Eurasian Blackbird | *Turdus merula* | **Europe 3** | Würzburg (BY) |
| 2018 | MK956009 | Great Grey Owl | *Strix nebulosa* | **Africa 3** | Nordhorn (LS) |
| 2018 | MK956011 | Great Grey Owl | *Strix nebulosa* | **Europe 3** | Hannover (LS) |
| 2018 | MK956012 | Northern hawk-owl | *Surnia ulula* | **Africa 3** | Wingst (LS) |
| 2018 | MK956014 | Great Grey Owl | *Strix nebulosa* | **Africa 3** | Osnabrück (LS) |
| 2018 | MK956019 | Great Grey Owl | *Strix nebulosa* | **Europe 3** | Rosengarten (LS) |
| 2018 | MK956003 | Eurasian Blackbird | *Turdus merula* | **Europe 3** | Brietlingen (LS) |
| 2018 | MK956004 | Eurasian Blackbird | *Turdus merula* | **Europe 3** | Osterholz-Scharnbeck (LS) |
| 2018 | MK956005 | Eurasian Blackbird | *Turdus merula* | **Europe 3** | Lüneburg (LS) |
| 2018 | MK956016 | Great Grey Owl | *Strix nebulosa* | **Africa 3** | Halle (ST) |
| 2018 | MK956055 | Eurasian Blackbird | *Turdus merula* | **Europe 3** | Dresden (SN) |
| 2018 | MK956006 | Great Grey Owl | *Strix nebulosa* | **Africa 3** | Gera (TH) |
| 2018 | MK956007 | Eurasian Blackbird | *Turdus merula* | **Europe 3** | Freigericht (HE) |
| 2018 | MK956008 | Eurasian Blackbird | *Turdus merula* | **Europe 3** | Gießen (HE) |
| 2018 | MK956041 | Eurasian Blackbird | *Turdus merula* | **Europe 3** | Aumühle (SH) |
| 2018 | MK956042 | Eurasian Blackbird | *Turdus merula* | **Africa 3** | Brickeln (SH) |
| 2018 | MK956043 | Eurasian Blackbird | *Turdus merula* | **Africa 3** | Lübeck (SH) |
| 2018 | P 819 (sequence identical to MK956004) | Eurasian Blackbird | *Turdus merula* | **Europe 3** | Joldelund (SH) |
| 2018 | MK956023 | Eurasian Blackbird | *Turdus merula* | **Africa 3** | Bergen/Rügen (MV) |
| 2018 | MK956024 | Eurasian Blackbird | *Turdus merula* | **Africa 3** | Grevesmühlen (MV) |
| 2018 | MK956025 | Eurasian Blackbird | *Turdus merula* | **Europe 3** | Parchim (MV) |
| 2018 | MK956026 | Eurasian Blackbird | *Turdus merula* | **Europe 3** | Warin (MV) |
| 2018 | MK956027 | Eurasian Blackbird | *Turdus merula* | **Africa 3** | Prohn (MV) |
| 2018 | MK956022 | Song Thrush | *Turdus philomelos* | **Europe 3** | Berlin (BE) |
| 2018 | VL 56389 (sequence identical to MK956046) | Eurasian Blackbird | *Turdus merula* | **Africa 3** | Chemnitz (SN) |
| 2018 | VL 59686 (sequence identical to MK956044 | Eurasian Blackbird | *Turdus merula* | **Europe 3** | Mittweida (SN) |
| 2018 | VL 60006 (sequence identical to MK956045) | Eurasian Blackbird | *Turdus merula* | **Europe 3** | Freiberg (SN) |
| 2018 | VL 61470 (sequence identical to MK956046) | Eurasian Blackbird | *Turdus merula* | **Europe 3** | Chemnitz (SN) |

SN = Saxony, ST = Saxony-Anhalt, BE = Berlin, HE = Hesse, NRW = North Rhine-Westphalia, LS = Lower Saxony, SH = Schleswig-Holstein, BW = Baden-Wurttemberg, SL = Saarland, BY = Bavaria, MV = Mecklenburg-Western Pomerania, TH = Thuringia
